# Upper gastrointestinal bleeding on veno-arterial extracorporeal membrane oxygenation support

**DOI:** 10.1186/s13613-024-01326-x

**Published:** 2024-07-03

**Authors:** Quentin de Roux, Yekcan Disli, Wulfran Bougouin, Marie Renaudier, Ali Jendoubi, Jean-Claude Merle, Mathilde Delage, Lucile Picard, Faiza Sayagh, Chamsedine Cherait, Thierry Folliguet, Christophe Quesnel, Aymeric Becq, Nicolas Mongardon

**Affiliations:** 1 Service d’anesthésie-réanimation et médecine péri-opératoire, DMU CARE, DHU A-TVB, Assistance Publique-Hôpitaux de Paris, Hôpitaux Universitaires Henri Mondor, Créteil, France; 2 Réanimation polyvalente, Ramsay Générale de Santé, Hôpital Privé Jacques Cartier, Massy, France; 3 Service de chirurgie cardiaque, Assistance Publique-Hôpitaux de Paris, DMU CARE, Hôpitaux Universitaires Henri Mondor, Créteil, France; 4 Service de gastro-entérologie, Assistance Publique-Hôpitaux de Paris, Hôpitaux Universitaires Henri Mondor, Créteil, France; 5 Faculté de Santé, Université Paris Est Créteil, Créteil, France; 6 U955-IMRB, Equipe 03 “Stratégies pharmacologiques et thérapeutiques expérimentales des insuffisances cardiaques et coronaires”, Inserm, UPEC, Ecole Nationale Vétérinaire d’Alfort, Maisons-Alfort, France; 7 AfterROSC research group, Paris, France; 8 Service d’Anesthésie-Réanimation et Médecine Péri-Opératoire, CHU Henri Mondor, 1 rue Gustave Eiffel, Créteil, 94000 France

## Abstract

**Introduction:**

Patients on veno-arterial extracorporeal membrane oxygenation (V-A ECMO) support are at a high risk of hemorrhagic complications, including upper gastrointestinal bleeding (UGIB). The objective of this study was to evaluate the incidence and impact of this complication in V-A ECMO patients.

**Materials and methods:**

A retrospective single-center study (2013–2017) was conducted on V-A ECMO patients, excluding those who died within 24 h. All patients with suspected UGIB underwent esophagogastroduodenoscopy (EGD) and were analyzed and compared to the remainder of the cohort, from the initiation of ECMO until 5 days after explantation.

**Results:**

A total of 150 V-A ECMO cases (65 after cardiac surgery and 85 due to medical etiology) were included. 90% of the patients received prophylactic proton pump inhibitor therapy and enteral nutrition. Thirty-one patients underwent EGD for suspected UGIB, with 16 confirmed cases of UGIB. The incidence was 10.7%, with a median occurrence at 10 [7–17] days. There were no significant differences in clinical or biological characteristics on the day of EGD. However, patients with UGIB had significant increases in packed red blood cells and fresh frozen plasma needs, mechanical ventilation duration and V-A ECMO duration, as well as in length of intensive care unit and hospital stays. There was no significant difference in mortality. The only independent risk factor of UGIB was a history of peptic ulcer (OR = 7.32; 95% CI [1.07–50.01], *p* = 0.042).

**Conclusion:**

UGIB occurred in at least 1 out of 10 cases of V-A ECMO patients, with significant consequences on healthcare resources. Enteral nutrition and proton pump inhibitor prophylaxis did not appear to protect V-A ECMO patients. Further studies should assess their real benefits in these patients with high risk of hemorrhage.

**Supplementary Information:**

The online version contains supplementary material available at 10.1186/s13613-024-01326-x.

## Introduction

Refractory cardiogenic shock is associated with high short-term mortality [1–3]. Despite optimal etiological and symptomatic management, persistent or worsening organ failure may lead to temporary extracorporeal circulatory support by veno-arterial extracorporeal membrane oxygenation (V-A ECMO), until cardiac function recovery, long-term circulatory support implantation or heart transplantation. Regardless of the etiology of cardiogenic shock, V-A ECMO is associated with specific morbidity [4, 5]. The most frequent adverse events of extracorporeal life supports are thrombotic complications and hemorrhagic syndromes [6]. Among the latter, external or internal bleedings may be favored by curative anticoagulation, acquired von Willebrand syndrome, thrombocytopenia, platelet dysfunction or hypofibrinogenemia [7, 8]. An excessive endogenous fibrinolysis is also an emerging mechanism [9, 10].

In critically ill patients, upper gastrointestinal bleeding (UGIB) is a frequent complication. Erosions of the gastric mucosa and subepithelial microhemorrhages are found in 75–100% of these patients [11]. Studies report an incidence of clinically significant UGIB (i.e., requiring treatment) in 0.6–4% of critically ill patients [12]. UGIB has negative impact on the outcome of critically ill patients, as well as increases the length of stay in intensive care unit (ICU) [13]. However, interventions and studies on digestive hemorrhage did not target patients receiving V-A ECMO support, a population that is particularly at risk in this respect.

For patients with UGIB on ECMO, the literature is sparse, covering all kinds of bleeding complications, involving a heterogeneous population (pediatric or adult), grouping patients with veno-venous (V-V) and V-A ECMO and making no distinction between upper and lower digestive tract bleedings. In addition, information on clinical management and the way by which gastrointestinal bleeding has been diagnosed is partial [14–16].

Firstly, we conducted this study to determine the incidence of UGIB in patients receiving V-A ECMO for refractory cardiogenic shock. Secondly, we aimed at identifying the risk factors associated with UGIB and assessing the link of this event with patient outcomes. We hypothesized that its complication was rare and was associated with increased morbidity without affecting mortality.

## Materials and methods

### Study setting and population

This is a retrospective monocentric study carried out in the cardiovascular surgical ICU at Henri Mondor University Hospital (1,000-bed tertiary hospital in Créteil, France).

All patients requiring V-A ECMO support from January 2013 to January 2017 were included. Amongst these patients, those over 18 years of age receiving peripheral (femoral-femoral) V-A ECMO for refractory cardiogenic shock of medical etiology (including refractory cardiac arrest or post-cardiac arrest shock) or secondary to failure to wean off extracorporeal circulation after cardiac surgery were included. Patients requiring central V-A ECMO or patients who died during the first 24 h after V-A ECMO implantation were excluded.

### Implantation and weaning of veno-arterial ECMO

Indications and weaning of V-A ECMO followed the recommendations for the management of cardiogenic shock that was considered refractory despite optimization of medical treatment [17–19]. In the case of acute cardiogenic shock or post-cardiotomy cardiogenic refractory shock, or refractory cardiac arrest, peripheral V-A ECMO was implanted. Initial flow was set at 50–65 mL/kg/min and adjusted according to clinical and biological findings. Norepinephrine dose was adjusted to target a mean arterial pressure of 65–70 mmHg [20, 21]. Vasopressin was not used. Withdrawal was performed according to standard recommendations [19].

### Definitions of upper gastro-intestinal bleeding and endoscopic procedures

UGIB was suspected in case of melena, bright red blood per rectum or hematemesis and/or acute anemia, with or without hyperuremia, or in case of unexplained hemodynamic instability. In every one of these scenarios, a bedside esophagogastroduodenoscopy (EGD) was performed in the ICU by the digestive endoscopy team, except in cases of moribund patient. UGIB was confirmed if a causative lesion was found (active bleeding or lesions that may be responsible for bleeding but are no longer bleeding) [22]. Lesions considered likely to be responsible for UGIB were ulcer, gastritis, gastric tube ulceration or esophagitis. Endoscopic treatment was left to the discretion of the physician performing the endoscopy, depending on the lesions found. The occurrence of EGD-confirmed UGIB while on V-A ECMO was recorded from the time the mechanical support was placed until five days after explantation, considering that the period after ECMO removal was still associated with significant complications and particularly exposed to ECMO-related hemorrhagic complications with ongoing anticoagulation [21, 23].

### Ulcer prophylaxis, nutrition, anticoagulation and transfusion support

Curative anticoagulation with continuous infusion of unfractionated heparin was prescribed, with an anti-Xa target of 0.3–0.6 IU/mL, in patients on V-A ECMO. Anticoagulation was initiated 24 h after ECMO placement, in the absence of bleeding syndrome [24]. Antiplatelet therapy was started on day 1 if indicated and in the absence of contraindication.

Enteral nutrition was introduced from day 2 onwards with a target of 20–25 kcal/kg/day at 5 days [25]. In the event of intolerance, parenteral nutrition was preferred.

According to our institutional protocol, prophylactic intravenous proton pump inhibitor (PPI) (pantoprazole 40 mg/day) was recommended in case of risk factors such as history of peptic ulcer, acute renal failure, indication for double antiplatelet therapy or curative anticoagulation, or absence of enteral feeding [22, 26].

If UGIB was suspected, medical treatment was started prior to the EGD. It consisted of discontinuing curative anticoagulation and antiplatelet therapy, correcting potential hemostasis disorders and reinforcing PPI treatment (80 mg IV bolus dose of pantoprazole followed by a continuous infusion at 8 mg/hour). Intravenous sandostatin was added if portal hypertension was suspected.

The transfusion threshold for packed red blood cells was set at 7 g/dL, and for platelet units at 50 G/L [27]. In bleeding situations, the targeted objective was to achieve a platelet count of 80 G/L³, a prothrombin time (PT) of over 70% and a plasma fibrinogen concentration of at least 1.5 g/L [28, 29].

### Data collection

Data were collected retrospectively from medical records. The etiology of cardiogenic shock, the initial need for cardiopulmonary resuscitation and its duration, the number of packed red blood cells, platelet concentrates and total fresh-frozen plasma administered during the ICU stay and the need for renal replacement therapy were recorded. Data on nutrition and time to initiation were also recorded. Vasoactive-Inotropic Score (VIS) was calculated as: dobutamine dose (µg/kg/min) + 100× epinephrine dose (µg/kg/min) + 100× norepinephrine dose (µg/kg/min) [30]. On the day of suspected UGIB and EGD, the time between EGD and ECMO initiation, the use of antiplatelet therapy in the 5 days prior to EGD, PPI therapy and effective anticoagulation were noted. Clinical data leading to the suspicion of UGIB and subsequent medical or endoscopic treatment were also described.

### Regulatory requirements

In accordance with French legislation, patients were informed of the anonymous extraction of data and the analysis of their records [31]. The Comité d’Ethique de la Recherche en Anesthésie-Réanimation approved this study (CERAR: IRB 00010254-2019-027), and the database was declared to the French Data Protection Authority (CNIL). This manuscript adheres to the STROBE guidelines [32].

### Statistical analyses

Continuous variables were expressed as median and interquartile range [25–75%] or mean (standard deviation), as appropriate. Missing data are available in supplementary material 1. Categorical variables were expressed in terms of numbers and percentages. Firstly, we compared patients with confirmed UGIB (i.e., EGD positive with a causative lesion) and patients without confirmed UGIB (i.e., EGD performed but no causative lesion, or no EGB performed in the absence of suspected bleeding) in univariate analysis applying chi-squared test for categorical variables, and Mann-Whitney test for continuous variables. Secondly, among patients with EGD performed, we compared patients with positive and negative EGD. A multivariable analysis, using backward selection, was subsequently performed to assess independent risk factors for confirmed UGIB, including variables with *p*-values < 0.15 in univariate analysis (2 variables: history of peptic ulcer and vasoactive-inotropic score), or with clinical relevance (2 variables: PPI prophylaxis and enteral nutrition). Considering the limited number of events, this multivariable analysis was performed for exploratory purpose only. Missing data were handled using case-complete analysis. All tests were two-sided, with a two-sided alpha risk of 5%. STATA/SE 14.0 software (College Station, TX, USA) was used for all analyses.

## Results

**Fig. 1:**
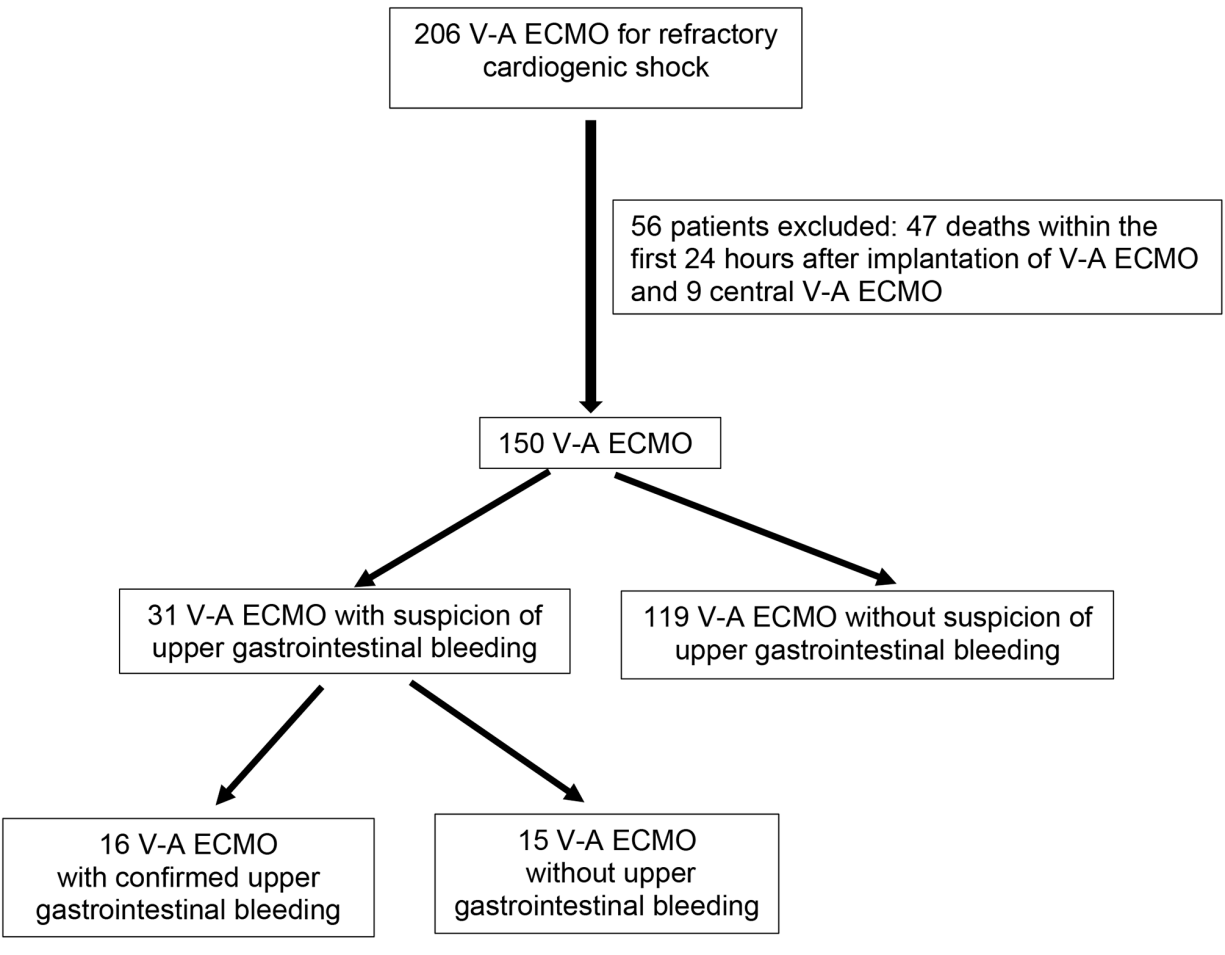
Flow chart

During this four-year period, 206 consecutive patients with V-A ECMO were managed in the ICU. After excluding 56 cases (nine patients with central support and 47 patients who died within 24 h of implantation), 150 V-A ECMO were included for analysis (Fig. 1). Patients receiving V-A ECMO twice during their ICU hospitalization (*n* = 3) were considered as independent cases.

### Description of the population

The median age was 58 years (48–69), with a majority of men (71%). The indications for assistance were post-cardiotomy cardiogenic shock (*n* = 65) or cardiogenic shock of medical origin (*n* = 85); more than a quarter of them were patients with refractory cardiac arrest or post-cardiac arrest shock (26%). V-A ECMO support was initiated at 0 (0–1) days after ICU admission. Table 1 shows patient comorbidities as well as clinical and biological characteristics at the time of V-A ECMO initiation. There was no significant difference in the demographic characteristics of patients with or without UGIB. Similarly, there was no significant difference in the occurrence of new shock, de novo atrial fibrillation, maximum norepinephrine dosage or the need for renal replacement therapy during the 5 days following V-A ECMO initiation.

**Table 1:** Patient characteristics at admission, during the five days after V-A ECMO support and on the day of EGD

Characteristics	General population (*n* = 150)	Patient with UGIB (*n* = 16)	Patient without UGIB (*n* = 134)	*P*
***Baseline characteristics***				
Age (years)	58 [48–69]	59 [48–76]	58 [48–69]	0.58
Male sex	104 (71)	10 (62)	94 (70)	0.57
SAPS II	55 [38–70]	48 [38–58]	53 [38–70]	0.24
BMI (kg/m^2^)	25 [23–29]	26 [23–29]	25 [23–29]	0.91
Smoker	51 (35)	5 (31)	46 (35)	0.99
Diabetes	36 (24)	2 (12)	34 (25)	0.36
Peripheral arterial disease	8 (5)	0 (0)	8 (6)	0.60
Arterial hypertension	63 (42)	6 (37)	57 (42)	0.70
Chronic alcoholism	8 (5)	2 (12)	6 (4)	0.20
Cirrhosis	3 (2)	1 (6)	2 (1)	0.28
History of ulcer	5 (3)	2 (12)	3 (2)	0.08
***At time of V-A ECMO initiation***				
Indication for V-A ECMO				
*Post-operative cardiac surgery	65 (43)	7 (43)	58 (43)	0.97
*Medical etiology	85 (57)			
*Including refractory cardiac arrest or post-cardiac arrest shock*	39 (26)	5 (31)	34 (25)	0.56
Vasoactive-Inotropic Score	70 [34–140]	43 [14–145]	70 [34–140]	0.14
SOFA at the day of ECMO initiation	9 [5–13]	9 [5–12]	9 [5–13]	0.98
Arterial lactate (mmol/L)	4 [2.3–6.6]	3.25 [2.3–7.05]	4 [2.3–6.6]	0.76
Serum creatinine (µmol/L)	124 [92–195]	109 [87–132]	124 [92–195]	0.16
Hemoglobin (g/dL)	11.2 [9.3–13.0]	11.9 [9.8–13.5]	11.2 [9.3–13.0]	0.30
Platelet count (G/L)	186 [123–249]	199 [123–263]	186 [123–249]	0.90
Prothrombin level (%)	54 [44–70]	62 [45–80]	54 [44–70]	0.26
***Events during five days after V-A ECMO initiation***				
Septic shock	37 (25)	2 (22)	25 (33)	0.71
Hemorrhagic shock	42 (28)	3 (20)	39 (29)	0.55
De novo atrial fibrillation	13 (9)	0 (0)	13 (10)	0.36
Renal replacement therapy	47 (31)	4 (26)	43 (32)	0.77
Maximum dose of norepinephrine (µg/kg/min)	1.00 [0.47–2.0]	0.90 [0.4–2.2]	1.05 [0.47–2.0]	0.74
Enteral nutrition	128 (88)	15 (93)	113 (87)	0.69

Data are expressed as median [interquartile 25–75] or number (percentage), as appropriate

Abbreviations: V-A ECMO: veno-arterial extracorporeal membrane oxygenation; BMI: body mass index; SAPS 2: Simplified Acute Physiology Score 2; SOFA: sequential organ failure assessment; EGD: esophagogastroduodenoscopy; PPI: proton pump inhibitor

In the 31 patients with suspected UGIB, EGD was systematically performed and confirmed the presence of lesions compatible with UGIB in 16 patients (52%). On the day of the EGD, compared with the general population, the 16 patients with a positive EGD were not significantly more treated with anticoagulants, antiplatelet therapies, PPIs and received enteral nutrition in a similar proportion (Table 1).

### Suspicion of upper gastrointestinal bleeding and diagnosis

UGIB was suspected in 31 patients on V-A ECMO (21%). The clinical symptoms (blood loss) arising suspicion of UGIB and biological characteristics at the time of EGD are detailed in Table 2. Some patients could have one or more of signs leading to the suspicion of UGIB. UGIB confirmation was positive in 16 patients, revealing at least one of these lesions: gastritis (*n* = 8), feeding tube ulceration (*n* = 8), esophagitis (*n* = 7) or peptic ulcer (*n* = 3). Ischemic lesions were found in five patients. Three cases of bezoars were reported. No patient had endoscopic signs of portal hypertension. Of the 16 patients, six received specific hemostatic therapies such as clip placement (*n* = 3) and topical hemostatic powder (*n* = 3). Six patients had more than one endoscopy because of recurrent UGIB. No patient required radiological or surgical hemostasis.

**Table 2:** Clinical blood loss manifestations and biological data of patients on the day of esophagogastroduodenoscopy

Characteristics	Patient with positive EGD (*n* = 16)	Patient with negative EGD (*n* = 15)	*p*
Melena	6 (38)	2 (13)	0.22
Hematemesis	4 (25)	2 (13)	0.65
Bright red bleeding per rectum	1 (6)	3 (20)	0.33
Time between V-A ECMO initiation and EGD	10 [7–17]	6 [3–9]	0.03
PPI prophylaxis	14 (87)	13 (93)	0.99
Anticoagulant treatment	11 (69)	7 (50)	0.29
Antiplatelet treatment	9 (60)	7 (50)	0.58
Platelet count (G/L)	81 [46–13]	76 [45–119]	0.82
Prothrombin time (%)	77 [64–82]	68 [64–75]	0.24
Anti-Xa activity (IU/mL)	0.27 [0.10–0.33]	0.10 [0.10–0.25]	0.18

PT, anti-Xa activity and platelet count did not significantly differ between patients with positive and negative EGD. Median time between ECMO initiation and EGD performance was significantly longer in case of positive EGD (10 [7–17] vs. 6 [3–9] days, *p* = 0.03).

### Outcomes of patients with confirmed upper gastrointestinal bleeding

Patient outcomes are presented in Table 3. Mortality in patients with confirmed UGIB was similar for patients without UGIB (*p* = 0.98). They were not more likely to require renal replacement therapy. Patients with positive EGD had a significantly higher number of packed red blood cells transfused compared with patients without UGIB (27 [21–37] vs. 14 [8–25], *p* < 0.001). Similarly, the number of units of transfused platelet was also significantly higher in the UGIB group (3 [2–8] vs. 2 [1–4], *p* = 0.01); however, the number of units of transfused fresh frozen plasma was not significantly different.

**Table 3:** Healthcare resources consumption and outcomes during ICU course of patients with and without upper gastrointestinal bleeding

Characteristics	General population (*n* = 150)	Patient with positive EGD (*n* = 16)	Patient with negative EGD or without EGD (*n* = 134)	*p*
Total number of packed red blood cells transfused	14 [8–2]	27 [21–37]	14 [8–25]	< 0.001
Total number of platelet concentrates units transfused	2 [1–4]	3 [2–8]	2 [1–4)	< 0.01
Total number of fresh frozen plasmas units transfused	6 [3–12]	8 [4–17]	6 [3–12)	0.07
Renal replacement therapy	73 (49)	11 (69)	62 (46)	0.075
Duration of V-A ECMO support (days)	7 [5–13]	17 [10–20]	7 [5–13]	< 0.001
Duration of mechanical ventilation (days)	14 [6–27]	30 [18–52]	14 [6–27]	0.001
ICU length of stay (days)	19 [10–32]	28 [20–62]	18 [10–32]	< 0.001
Hospital length of stay (days)	24 [14–38]	34 [22–74]	24 [14–38]	0.002
Mortality	84 (56)	9 (56)	75 (56)	0.98

The duration of V-A ECMO support, mechanical ventilation, ICU length of stay and hospital length of stay were also significantly longer in patients with confirmed UGIB.

In multivariate analysis (Table 4), the only independent factor associated with the occurrence of UGIB was a prior history of peptic ulcer disease (OR 7.32; 95% CI [1.07; 50.01], *p* = 0.042). The introduction of enteral nutrition or PPI prophylaxis within five days of ECMO implantation was not significantly associated with UGIB occurrence.

**Table 4:** Independent factors associated with the occurrence of upper gastro-intestinal bleeding

	OR [95% CI]	*p*
History of peptic ulcer	7.32 [1.07; 50.01]	0.04
Enteral nutrition within 5 days of V-A ECMO implantation	2.16 [0.25; 18.99]	0.48
PPI prophylaxis within 5 days of V-A ECMO implantation	0.45 [0.11; 1.86]	0.27

## Discussion

In this study of 150 consecutive V-A ECMO, confirmed UGIB occurred in at least 1 out of 10 patients after a median delay of 10 days after V-A ECMO implantation. Only one third of EGDs led to hemostatic procedures. Patients with confirmed bleeding received almost twice as many transfusions of packed red blood cells and fresh frozen plasma units during their ICU stay. While mortality was not significantly different between patients with and without UGIB, healthcare resources were largely involved, with longer ICU and hospital lengths of stay as well as longer duration of V-A ECMO support and mechanical ventilation in the UGIB group. The only independent risk factor for the occurrence of UGIB was a history of peptic ulcer disease.

Digestive complications on V-A ECMO have been scarcely investigated. Previously, our research group deciphered the rare but dramatic issue of acute mesenteric ischemia on V-A ECMO [20].

Here, we further expanded this area of research by studying V-A ECMO-related UGIB. Surprisingly, only a few studies have looked specifically at the occurrence of gastrointestinal bleeding while on V-A ECMO [14–16]. A single-center retrospective cohort study found a 13.6% incidence of gastrointestinal bleeding, with a time to onset of 8 days [8]. The conclusions were limited by the fact that 64 V-V ECMO were included amongst 132 patients. In addition, the definition of gastrointestinal hemorrhage was broader, as it took into account all externalizations of blood. Thus, only 7 of the 18 patients with externalized blood loss had an EGD, and 50% of patients had no diagnosis for this gastrointestinal hemorrhage, which could be located in the upper or lower digestive tract. In a recent retrospective cohort including 455 V-A ECMO patients, a 10% incidence of UGIB with an onset time of 12 days was found [14]. The definition of UGIB was broader than ours, as it also included patients who presented with externalized bleeding (hematemesis, melena), but with no EGD performed. Our study confirms the incidence and the time to onset of UGIB on V-A ECMO. To note, this incidence is probably underestimated as moribund patients were excluded and as EGD was not systematic. The strength of our UGIB definition lies in the fact that the symptomatology giving rise to suspicion had to be confirmed by EGD, which is the gold standard test to confirm UGIB [15].

Importantly, our study adds to the current literature on UGIB in patients on V-A ECMO by specifically investigating nutrition modalities, a major data in digestive complications which was overlooked in previous studies. We also analyzed medical and biological conditions on the day of UGIB diagnosis. In the work carried out here, a history of peptic ulcer was the only independent risk factor for the occurrence of UGIB in patients undergoing V-A ECMO, a parameter which has clinical sound and statistical significance despite wide confidence interval. In the single study close to ours, the risk factors were a history of peptic ulcer, double antiplatelet therapy and extracorporeal cardiopulmonary resuscitation [14]. While our study is the second one to suggest that peptic ulcer history promotes UGIB, the two other risk factors were not identified herein, perhaps due to lack of power. However, while the previous studies displayed data at the time of ICU admission and cannulation, none had information on parameters related to patient characteristics on the day of bleeding suspicion/EGD, such as the presence or absence of anticoagulant overdose, the existence of hemostasis disorders, prothrombin time, hemoglobin and platelet levels. Additionally, no data regarding nutrition modalities was available. In contrast, data on route and timing were collected in our study, and none of the biological data differed, which suggests that coagulation disorders related to ECMO or its associated treatments did not influence the occurrence of UGIB. We also investigated new and dynamic parameters, i.e. the impact of a new shock after implantation of the support, the cumulative dose of vasopressor/inotropic drugs via the vasoactive-inotropic score (VIS score), the presence of antiplatelet therapy in the 5 days prior to suspected UGIB, the presence of ulcer prophylaxis on the day of suspicion, and the delay between start of enteral nutrition and implantation of the assistance. These parameters appears to be factors of tissue aggression, and possibly risk factors for UGIB [14, 33]. However, early enteral nutrition may increase mortality risk in critically ill patients and digestive ischemia in patients with shock [34, 35]. In the NUTRIREA-2 study, which compared early parenteral nutrition with early enteral nutrition in intensive care patients, there was no significant difference in mortality or infectious complications, but a significant increase was found in digestive complications such as diarrhea, vomiting and digestive ischemia in the enteral group patients [36]. To note, UGIB or onset of gastro-duodenal ulcer were not specifically investigated in this study [32]. These data raise questions about the interest and timing of enteral nutrition in the specific subgroup of patients on V-A ECMO. Indeed, enteral nutrition could have appeared to be a protective factor by promoting intestinal trophicity, as early initiation of enteral nutrition (in the first 5 days of ECMO support) was negatively associated with mesenteric ischemia on V-A ECMO [20]. So, there is no evidence suggesting that early enteral nutrition could be harmful in patients on V-A ECMO [37]. It is interesting to note that in the NUTRIREA-3 study comparing low versus standard calorie and protein intake in critically ill patients, there were less mesenteric ischemia in the low target group [38]. However, we may speculate that the weight of nutrition modalities may be lower than ulcer history in the occurrence of UGIB.

90% of the patients in this cohort had received PPI prophylaxis and enteral nutrition, started within 5 days of ECMO implantation. In our logistic regression analysis, these factors were not negatively associated with the occurrence of UGIB. In the literature, the interest of ulcer prophylaxis remains debated, with one meta-analysis finding a significant reduction in UGIB events [39]. Other meta-analyses found a significant reduction in all types of bleeding but an increase in complications such as ventilator-associated pneumonia [40, 41]. Recently, the SUP-ICU randomized controlled trial investigating the value of systematic ulcer prophylaxis with PPIs in critically ill patients demonstrated no improvement of survival at day 90 [42]. Although the incidence of UGIB in ICU is less than 5%, no study comparing patients on and off ECMO is available to isolate specific risk factors of bleeding.

Beyond the inherent limitations of a single-center retrospective study, certain limitations should be addressed. Firstly, patients on central V-A ECMO were excluded from our analysis because of their low number (*n* = 9, versus 150 peripheral V-A ECMO), creating heterogeneity and making subgroup analysis impossible. In addition, central cannulation is restricted to post-cardiotomy patients, making it much more rare than peripheral one. Secondly, although ICU and surgical teams were unchanged during the inclusion period of four years, with unchanged protocols, the design of the study did not allow us to ascertain the impact of time on potential changes in practices and management. Despite the robustness of UGIB definition, some patients with UGIB symptoms may not have benefited from an endoscopic exploration due to the severity of their clinical condition. Similarly, patients who died within the first twenty-four hours were not included and may have had compatible lesions and symptoms, potentially increasing the real incidence of UGIB. Thirdly, while most of our patients had PPI prophylaxis through a dedicated protocol, we had no data on the dosage, drug or administration route of the treatment. Similarly, despite enteral nutrition protocol, with almost 9 out of 10 patients receiving enteral nutrition five days after V-A ECMO initiation, the achievement of the calorie target was not assessed. Fourthly, certain pathophysiological mechanisms, such as platelet dysfunction, acquired von Willebrand disease, hypofibrinogenemia and hyperfibrinolysis may be observed during V-A ECMO support and increase the risk of bleeding events, but they are not assessed routinely and were not evaluated here [43–46]. In the absence of fibrinogen levels and D-dimers measurements, the respective influence of these factors could not be deciphered. To note, our protocol was to target an anti-Xa level of 0.3–0.6 U/mL with unfractioned heparin, but the most recent guidelines suggest achieving an anti-Xa level of 0.3–0.5 U/mL [47]. As the observed anti-Xa levels were at the lower levels of this target, this difference of practices had presumably a poor impact on UGID incidence. As chronic anticoagulant therapies were not collected, one may also question the influence of previous medications on the risk hemorrhagic complications. However, all previous anticoagulant therapy had been stopped before the surgical procedure for patients with post-cardiotomy V-A ECMO support, in accordance with recommendations. In the case of medical patients, they may have had chronic medications that were still acting at the time of ECMO procedure. However, the time elapsed between ECMO insertion and the onset of bleeding (median onset of 10 days) suggests that previous chronic anticoagulant therapies were unlikely related to current bleeding complications. Fifthly, even though relevant data were detailed over the first 5 days of ECMO initiation, there is a window of time during which certain events may not have been detected given the fact that some patients had a UGIB afterwards. Finally, UGIB was associated with longer duration of V-A ECMO or ventilatory supports and longer duration of ICU length of stay. However, this association may also be related to the duration of exposure, that is longer ECMO exposure created more opportunities of hemorrhagic complications.

## Conclusion

Our study adds to the scarce literature describing digestive complications, and more specifically the occurrence of UGIB, in patients undergoing V-A ECMO for refractory cardiogenic shock. The incidence is higher than in the general population of critically ill patients. While mortality was unaffected, medical care consumption (blood transfusion and lengths of hospitalization) was much higher. The only risk factor was a history of peptic ulcer, despite early enteral nutrition and well-conducted PPI prophylaxis, which did not appear to protect patients on V-A ECMO. Further studies would be interesting to assess the real benefit of a more targeted ulcer prophylaxis.

### Electronic supplementary material

Below is the link to the electronic supplementary material.

Supplementary Material 1

## Data Availability

Data and materials are available on reasonable request to the corresponding author.
