# Supplementary material for: Upper gastrointestinal bleeding on veno-arterial extracorporeal membrane oxygenation support
Source: Ann Intensive Care. 2024 Jul 3;14:104. doi: 10.1186/s13613-024-01326-x (PMC11222359; doi:10.1186/s13613-024-01326-x)
Supplement: Supplementary file 1 — Supplementary Material 1 [file 13613_2024_1326_MOESM1_ESM.docx]

**Supplementary material 1**

| **Characteristics** | **Number of missing data** | **% (/150)** |
| --- | --- | --- |
| **EGD performed** | 0 | 0 |
| **UGIB among EGD** | 0/31 | 0 |
| **Age (years)** | 0 | 0 |
| **Male sex** | 0 | 0 |
| **SAPS II** | 0 | 0 |
| **BMI (kg/m^2^)** | 0 | 0 |
| **Smoker** | 3 | 2 |
| **Diabetes** | 0 | 0 |
| **Peripheral arterial disease** | 0 | 0 |
| **Cirrhosis** | 0 | 0 |
| **History of ulcer** | 0 | 0 |
| **Chronic alcoholism** | 0 | 0 |
| **Arterial hypertension** | 0 | 0 |
| **Indication for V-A ECMO**  *Post-operative cardiac surgery  *Medical etiology  *Including refractory cardiac arrest or post-cardiac arrest shock* | 0 | 0 |
| **Vasoactive-Inotropic Score** | 0 | 0 |
| **SOFA** | 0 | 0 |
| **Arterial lactate (mmol/L)** | 0 | 0 |
| **Serum creatinine (µmol/L)** | 22 | 15 |
| **Hemoglobin (g/dL)** | 0 | 0 |
| **Platelet level (G/L)** | 24 | 16 |
| **Prothrombin time (%)** | 42 | 28 |
| **Septic shock** | 1 | <1 |
| **Hemorrhagic shock** | 1 | <1 |
| **De novo atrial fibrillation** | 2 | 1 |
| **Renal replacement therapy** | 0 | 0 |
| **Maximum dose of norepinephrine**  **(µg/kg/min)** | 0 | 0 |
| **Enteral nutrition** | 5 | 3 |
| **Current anticoagulant treatment** | 1 | <1 |
| **Current antiplatelet therapy** | 2 | 1 |
| **Current PPI prophylaxis** | 1 | <1 |
| **Duration of V-A ECMO support (days)** | 1 | <1 |
| **Duration of mechanical ventilation (days)** | 5 | 3 |
| **ICU length of stay (days)** | 0 | 0 |
| **Hospital length of stay (days)** | 1 | <1 |
| **Mortality** | 0 | 0 |
